# Biological Heart Rate Reduction Through Genetic Suppression of Gα_s_ Protein in the Sinoatrial Node

**DOI:** 10.1161/JAHA.111.000372

**Published:** 2012-04-24

**Authors:** Patrick Lugenbiel, Alexander Bauer, Kamilla Kelemen, Patrick A. Schweizer, Rüdiger Becker, Hugo A. Katus, Dierk Thomas

**Affiliations:** From the Department of Cardiology, Medical University HospitalHeidelberg, Germany

**Keywords:** electrophysiology, gene therapy, heart failure, heart rate, sinoatrial node

## Abstract

**Background:**

Elevated heart rate represents an independent risk factor for cardiovascular outcome in patients with heart disease. In the sinoatrial node, rate increase is mediated by β_1_ adrenoceptor mediated activation of the Gα_s_ pathway. We hypothesized that genetic inactivation of the stimulatory Gα_s_ protein in the sinoatrial node would provide sinus rate control and would prevent inappropriate heart rate acceleration during β-adrenergic activation.

**Methods and Results:**

Domestic pigs (*n*=10) were evenly assigned to receive either Ad-small interfering RNA (siRNA)-Gα_s_ gene therapy to inactivate Gα_s_ or adenovirus encoding for green fluorescent protein (Ad-GFP) as control. Adenoviruses were applied through virus injection into the sinoatrial node followed by epicardial electroporation, and heart rates were evaluated for 7 days. Genetic inhibition of Gα_s_ protein significantly reduced mean heart rates on day 7 by 16.5% compared with control animals (110±8.8 vs 131±9.4 beats per minute; *P*<0.01). On β-adrenergic stimulation with isoproterenol, we observed a tendency toward diminished rate response in the Ad-siRNA-Gα_s_ group (Ad-siRNA-Gα_s_, +79.3%; Ad-GFP, +61.7%; *n*=3 animals per group; *P*= 0.294). Adverse effects of gene transfer on left ventricular ejection fraction (LVEF) were not detected following treatment (LVEF_Ad-siRNA-Gαs_, 66%; LVEF_Ad-GFP_, 60%).

**Conclusions:**

In this preclinical proof-of-concept study targeted Ad-siRNA-Gα_s_ gene therapy reduced heart rates during normal sinus rhythm compared with Ad-GFP treatment and prevented inappropriate rate increase after β-adrenergic stimulation. Gene therapy may provide an additional therapeutic option for heart rate reduction in cardiac disease. **(*J Am Heart Assoc*. 2012;1:jah3-e000372 doi: 10.1161/JAHA.111.000372)**

## Introduction

Elevated heart rate is increasingly recognized as a modifiable risk factor in patients with heart disease. Two recent randomized trials (morbidity–mortality evaluation of the *I*_f_ inhibitor ivabradine in patients with coronary disease and left ventricular dysfunction, BEAUTIFUL; systolic heart failure treatment with the *I*_f_ inhibitor ivabradine trial, SHIFT) identified a resting heart rate >70 beats per minute (bpm) as risk factor for cardiac outcome in patients with coronary artery disease and heart failure.^1–5^ Standard pharmacological management of patients with cardiovascular disease includes β_1_-selective blockers, exerting beneficial effects on morbidity and mortality that are mediated in part through heart rate-lowering properties. In a subset of patients, however, the heart rate remains elevated during β blocker treatment. In addition, adverse effects on intracardiac electrical conduction or on myocardial contractility may limit the use of β blockers. A specific inhibitor of the cardiac pacemaker current (*I*_f_) in the sinoatrial node (SAN), ivabradine, has been developed to provide heart rate reduction without affecting electrical conduction, cardiac contractility, or blood pressure. *I*_f_ is activated by membrane potential hyperpolarization and regulated by direct binding of cAMP in response to β-adrenergic stimulation.^6,7^ Pharmacological heart rate reduction with ivabradine improved cardiovascular outcome in BEAUTIFUL and SHIFT trial subgroups, confirming the significance of heart rate in cardiac disease.^1,3,4^

We sought to identify novel treatment modalities for heart rate reduction to further improve management and clinical outcome of heart failure patients. Targeted biological modification of cardiac electrophysiology may circumvent the disadvantage of non-specificity inherent to drug therapy. In particular, gene therapy has previously proven effective in preclinical proof-of-concept studies targeting atrial fibrillation.^8–13^ At the molecular level, β-adrenergic activation and subsequent increase of intracellular cAMP is mediated by Gα_s_ protein activation. We therefore hypothesized that genetic inactivation of the stimulatory Gα_s_ protein in the SA node would provide rate control during normal sinus rhythm and would prevent undesired heart rate acceleration during β-adrenergic activation. To test this hypothesis in a pilot study, an adenovirus encoding for a respective silencing RNA (Ad-siRNA-Gα_s_) was directly injected into sinoatrial nodes of domestic pigs, and heart rate was evaluated daily following gene transfer. Suppression of Gα_s_ subunits in the SAN significantly lowered heart rates compared with control animals treated with adenovirus encoding for green fluorescent protein (Ad-GFP). There were no adverse effects on systolic ventricular function. In addition, inappropriate rate increase was not observed after β-adrenergic stimulation with isoproterenol compared with Ad-GFP controls.

## Methods

### Adenoviruses

An adenovirus encoding for siRNA-Gα_s_ (Ad-siRNA-Gα_s_; SIRION, Martinsried, Germany) was used to suppress the stimulatory Gα_s_ protein. In the control group, a recombinant adenovirus encoding for green fluorescent protein (Ad-GFP; Qbiogen, Irvine, CA, USA), a reporter gene not affecting cardiac electrophysiology,^14,15^ was applied. Virus concentration was determined using a mouse antihexon antibody and horseradish peroxidase-conjugated goat anti-mouse secondary antibodies (Adeno-X Rapid Titer Kit, Clontech, Mountain View, CA, USA).

### HL-1 Cell Culture and In Vitro Gene Transfer

HL-1 cells, a cardiac muscle cell line derived from the AT-1 mouse atrial myocyte tumor lineage, were provided by Dr. William Claycomb (New Orleans, LA, USA).^16^ HL-1 cells were cultured and maintained as described previously.^13,16,17^ Gene transfer was performed when cells were 70% confluent by adding 0.2 mL solution containing Ad-GFP or Ad-siRNA-Gα_s_ (7.9×10^8^ plaque forming units) to 15 mL HL-1 culture media per 75 mL cell culture flask. Cells were harvested 48 hours after adenovirus application.

### Animals and In Vivo Gene Delivery

This study was approved by the Local Animal Care and Use Committee and has been carried out in accordance with the Guide for the Care and Use of Laboratory Animals as adopted and promulgated by the U.S. National Institutes of Health (NIH publication No. 86-23, revised 1985). The European Commission Directive 86/609/EEC and the current version of the German Law on the Protection of Animals was followed.

Domestic swine weighing 30 to 35 kg were investigated in this study. A single prophylactic dose of penicillin (200 mg; aniMedica, Senden-Bösensell, Germany) was administered before surgery. Pigs were sedated with ketamine (100 mg/kg; Roche, Grenzach-Wyhlen, Germany), anesthetized with propofol (1 mL of a 1% solution; Astra Zaneca, Wedel, Germany), and ventilated with isoflurane (1% to 2%; Baxter, Unterschleißheim, Germany) in a 1:2 ratio of O_2_ and N_2_O_2_. Ventilation, oxygenation, cardiac electrical activity, and body temperature were monitored and interdigital reflexes were tested to determine adequacy of anesthesia. Median thoracotomy was performed and the pericardium was opened to expose the heart under sterile conditions. Animals were randomized to receive either Ad-siRNA-Gα_s_ or Ad-GFP treatment. Then 1.5 mL solution containing Ad-siRNA-Gα_s_ (2×10^9^ plaque forming units) or Ad-GFP was injected in aliquots of 0.1 mL into the high right atrial wall, carefully avoiding injections into the atrial cavity. Injection of adenoviruses was followed by electroporation as reported previously.^10,12,13^ Five square wave applications were performed at the site targeted by gene therapy (20 V/100 ms; ECM 830, BTX Harvard Apparatus, Holliston, MA, USA). The electric field causes transient pores to form in the cells of the atrial tissue, improving adenovirus uptake into cells and resulting in ∼50% GFP transgene expression in pigs.^10^ After gene transfer and approximation of the pericardium the thorax was closed, and the animals received buprenorphine (0.324 mg; Essex Pharma, Munich, Germany) for 1 to 3 days after surgery. Heart rates reflect mean values obtained from two 6-lead ECG recordings performed daily during feeding. Animals were awake and alert at consistent levels during all ECG measurements during the observation period.

### β-Adrenergic Stimulation

Pharmacological studies were carried out in subgroups of *n*=3 pigs from each group on day 7. Isoproterenol (10 μg/kg; Sigma-Aldrich, Steinheim, Germany) was administered intravenously to sedated animals. Heart rate was continuously recorded using 6-lead ECG during the observation time. Baseline heart rate was recorded for 3 minutes before drug administration and for at least 10 minutes following isoproterenol application.

### Echocardiography

Echocardiography was performed on the day of gene transfer and before euthanization. Animals were sedated and anesthetized as described, and all examinations were carried out under similar conditions. A detailed description of echocardiographic analysis has been published previously.^13^

### Western Blot Analysis

After data acquisition on day 7, anesthetized animals were euthanized by intravenous application of KCl (1 M) and the hearts were removed and rinsed with phosphate buffered saline. Cardiac tissue was processed as described.^10,12,13^ HL-1 cells were solubilized for 1 hour at 4°C in lysis buffer containing 1% Triton X-100 and “Complete” protease inhibitors (Roche Diagnostics, Mannheim, Germany). Protein immunodetection was performed by sodium dodecyl sulfate gel electrophoresis and Western blotting as reported.^10,12,13^ Polyvinylidene difluoride membranes were developed by sequential exposure to blocking reagent (5% dry milk), primary antibodies directed against β_1_ adrenoceptor (sc-568; Santa Cruz Biotechnology, Heidelberg, Germany), Gα_s_ protein (sc-823; Santa Cruz Biotechnology), adenylate cyclase VI (sc-25500; Santa Cruz Biotechnology), phosphorylated protein kinase A (ab32390; Abcam, Cambridge, MA, USA), or glyceraldehyde-3-phosphate dehydrogenase (GAPDH; G8140-11, US Biological, Swampscott, MA, USA), and appropriate horseradish peroxidase-conjugated secondary antibodies (Abcam). Signals were developed using the enhanced chemiluminescence assay (GE Healthcare, ECL Western Blotting Reagents, Buckinghamshire, UK) and quantified using ImageJ 1.41 Software (National Institutes of Health, Bethesda, MD, USA). Protein content was normalized to GAPDH for quantification of optical density.

### Immunohistochemistry and Fluorescence Microscopy

Indicated tissue sections were processed and transgene efficiency was evaluated as described.^13^ For immunohistochemistry sections were incubated with polyclonal rabbit anti-Gα_s_ antibodies (sc-823; Santa Cruz Biotechnology). Antigen-antibody complexes were visualized with HRP-conjugated goat anti-rabbit IgG (7074; Cell Signaling, Danvers, MA, USA). Peroxidase activity was detected with diaminobenzidine (DAB) using the SK4100 kit (Vector Laboratories, Burlingame, CA, USA) according to the manufacturer's instructions. Direct (GFP) or indirect fluorescence (Gα_s_) was assessed using a fluorescence microscope (AX 70; Olympus, Hamburg, Germany). The percentage of cells exhibiting significant fluorescence signals compared with cells stained with 4,6-diamidino-2-phenylindole (Sigma-Aldrich) (direct fluorescence microscopy) or hematoxylin and eosin staining (immunohistochemistry) was quantified by blinded observers through cell counting in 5 (direct fluorescence) or 10 (immunodetection of Gα_s_ protein) randomly selected sections of each image.

### Statistics

**Figure 1.**
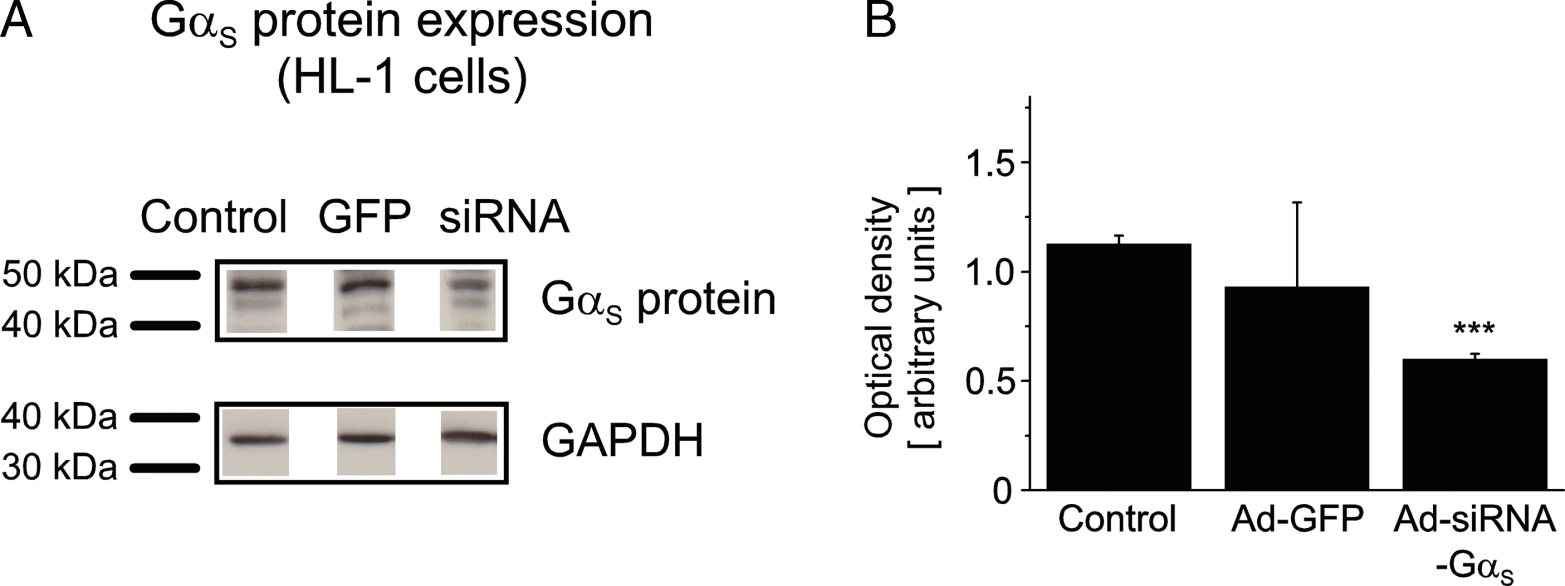
In vitro efficacy of Ad-siRNA-Gα_s_ gene transfer. Gα_s_ protein expression was analyzed using Western blot in HL-1 mouse atrial cardiac myocytes. (A) Gα_s_ protein levels evaluated in untreated control cells and following application of Ad-GFP (GFP) or Ad-siRNA-Gα_s_ (siRNA). (B) Quantification of optical density normalized to GAPDH protein. Gα_s_ expression was suppressed by 51% in HL-1 cells infected with Ad-siRNA-Gα_s_ (*n*=3) compared with controls (*n*=3), whereas Ad-GFP did not significantly alter Gα_s_ protein levels (*n*=3). Data are provided as mean±SEM; ***P<0.001 versus control HL-1 cells. GAPDH indicates glyceraldehyde-3-phosphate-dehydrogenase; GFP, green fluorescent protein.

Data are expressed as mean±SEM of *n* experiments. Normal distribution of the data was confirmed using the Shapiro-Wilk test (SPSS Statistics, IBM, Ehningen, Germany). We used unpaired or paired Student's *t* tests (two-tailed tests) to compare the statistical significance of the results where appropriate. *P*<0.05 was considered statistically significant. Heart rates were compared using two-factor analysis of variance (ANOVA) with treatment and time as factors and repeated measures on one factor (time). One-way ANOVA and Tukey's post hoc tests were then applied to identify paired differences between mean heart rates of treatment groups at different times. Multiple comparisons in Figure 1 were performed using one-way ANOVA. If the hypothesis of equal means could be rejected at the 0.05-level, pair wise comparisons of groups were made and the significance level was adjusted for multiple comparisons using the Bonferroni correction (0.05/*n*, where *n* is the number of tests performed). GFP expression in different cardiac regions was compared using repeated measures ANOVA and the Bonferroni post hoc test.

## Results

### In Vitro Efficacy of Ad-siRNA-Gα_s_ Gene Transfer

The efficacy of Gα_s_ protein suppression was analyzed in vitro in mouse atrial cardiac myocytes (HL-1 cells). An adenovirus transduction rate of 34% was previously reported under similar experimental conditions.^10^ Significant reduction of Gα_s_ protein in HL-1 cells was demonstrated by Western blot analysis 48 hours after Ad-siRNA-Gα_s_ treatment (−51.3%; *n*=3 independent assays; *P*=0.0005) compared with untreated HL-1 cells (Figure 1A, B). Ad-GFP application did not significantly affect Gα_s_ expression (*P*=0.643; *n*=3; Figure 1).

### Suppression of Gα_s_ Protein Provides Biological Heart Rate Reduction

**Figure 2.**
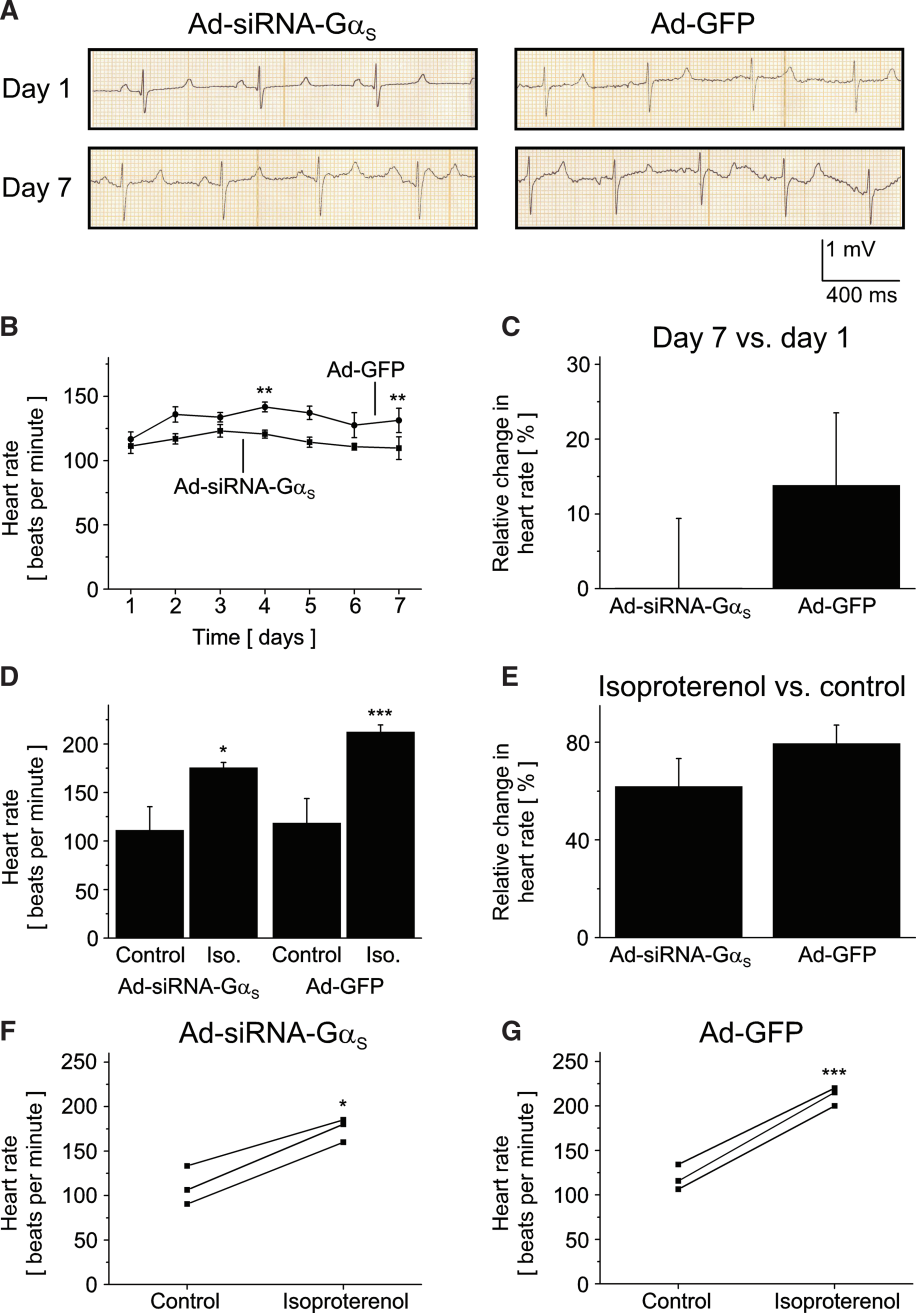
Heart rate reduction following Ad-siRNA-Gα_s_ gene therapy. (A) Representative ECG recordings obtained from pigs before gene therapy (day 1) and after application of Ad-siRNA-Gα_s_ or Ad-GFP (day 7), respectively. (B) Mean heart rates (± SEM) assessed by daily ECG recordings in control animals (*n*=5) and in pigs treated with Ad-siRNA-Gα_s_ (*n*=5). Statistical significances among groups were analyzed on days 1, 4, and 7, respectively (**P<0.01). (C) Relative changes in heart rates recorded on day 7 compared with the day of gene transfer (day 1). (D-G) Three animals from each group were subjected to isoproterenol challenge to assess adrenergic response. Administration of isoproterenol on day 7 significantly increased heart rates in Ad-GFP control animals (D, E). Heart rate acceleration by isoproterenol was attenuated in animals infected with Ad-siRNA-Gα_s_ (D, E). (F, G) Comparison of heart rates obtained from individual animals in the Ad-siRNA-Gα_s_ group (F) and in the Ad-GFP group (G) before and after isoproterenol challenge, respectively. Data represent mean values±SEM; *P<0.05, ***P<0.001 compared with respective drug-free control conditions. ECG indicates electrocardiogram; GFP, green fluorescent protein.

Ad-siRNA-Gα_s_ and Ad-GFP transfer was then performed in vivo using an established hybrid approach combining direct adenovirus injection into the sinoatrial node and epicardial electroporation to increase gene expression.^10,12,13^ Control animals treated with Ad-GFP (*n*=5) exhibited mean heart rates of 117±5.6 bpm before surgery and 131±9.4 bpm on day 7, respectively (Figure 2A, B). In contrast, pigs that received Ad-siRNA-Gα_s_ displayed mean heart rates of 111±5.6 bpm (day 1) and 110±8.8 bpm (day 7). Genetic inactivation of Gα_s_ protein reduced mean heart rates by 16.5% (day 7) compared with control pigs (*n*=5; *P*<0.01) (Figure 2A, B). During the entire follow-up period, the mean reduction of heart rates compared with the Ad-GFP group yielded 13.8±1.3% (range, 7.9% to 16.6%; corresponding to 10.6 to 22.8 bpm) (Figure 2B). Ad-GFP-infected control animals had a 13.8±9.7% (*n*=5; *P*=0.278) mean increase in heart rate when comparing day 7 with day 1 (Figure 2C) that was not statistically significant. This effect was not observed in Ad-siRNA-Gα_s_ pigs (+0.0±9.4%; *n*=5; *P*=0.882).

### Adrenergic Heart Rate Modulation

Activation of the sympathetic nervous system and subsequent heart rate increase enhance myocardial oxygen demand. In patients with heart disease, inappropriate heart rate acceleration after adrenergic stimulation increases the risk for myocardial ischemia and angina pectoris. Three animals from each group were subjected to isoproterenol application on day 7 to simulate activation of the β-adrenergic system. Heart rate was continuously monitored using 6-lead ECG during the observation time. Before drug administration, baseline heart rate was recorded for 3 minutes. Following drug application, heart rate was recorded for at least 10 minutes. For statistical evaluation, we determined peak effects after drug administration. β-adrenergic stimulation with isoproterenol increased heart rates by 79.3±7.7% in Ad-GFP control animals from 118±25.7 bpm to 212±7.6 bpm (*n*=3; *P*=0.0008; Figure 2D, E, G). In contrast, isoproterenol administration resulted in a 61.7±11.6% heart rate increase in the Ad-siRNA-Gα_s_ group (111±24.7 bpm vs 175±6.0 bpm; *n*=3; *P*=0.011; Figure 2D, E, F). The attenuated isoproterenol response in animals treated with Ad-siRNA-Gα_s_ did not reach statistical significance (*P*=0.294).

### Ad-siRNA-Gα_s_ Gene Therapy Did Not Affect Left Ventricular Function

**Figure 3.**
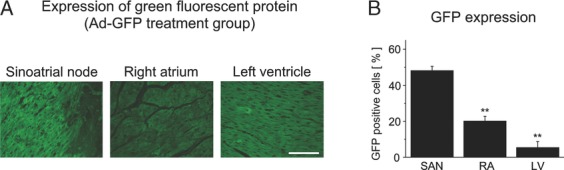
Efficacy and cardiac distribution of transgene expression. (A) Representative microphotographs depicting SAN, RA, and LV after application of Ad-GFP (day 7). GFP reporter gene expression was analyzed via direct fluorescence measurements (scale bar, 100 μm). (B) The relation of GFP positive cells compared with the total number of cardiac cells (in %) is presented for SAN, RA, and LV tissue obtained from 5 animals. Data are given as mean±SEM; **P<0.01 versus sinoatrial node. GFP indicates green fluorescent protein; LV, left ventricle; RV, right atrium; SAN, sinoatrial node.

Adenoviral gene transfer may exert adverse effects on cardiac function. To assess changes in left ventricular function, echocardiographic examinations were performed before gene transfer and after 7 days. Echocardiograms performed on day 1 revealed similar left ventricular ejection fractions (LVEF) among both study groups. Mean LVEF yielded 62.2±2.6% (Ad-siRNA-Gα_s_) and 61.1±2.3% (Ad-GFP), respectively (*n*=5 each; *P*=0.743). On the day of sacrifice, no reduction of LVEF was observed in study animals (LVEF_Ad-siRNA-Gαs_=62.5±2.4%; LVEF_Ad-GFP_=65.0±2.4%; *n*=5 each), consistent with low levels of transgene expression in left ventricles (see Figure 3). LVEF assessed on day 7 was not significantly different between treatment groups (*P*=0.479).

### In Vivo Gene Transfer Efficacy and Transgene Distribution

**Figure 4.**
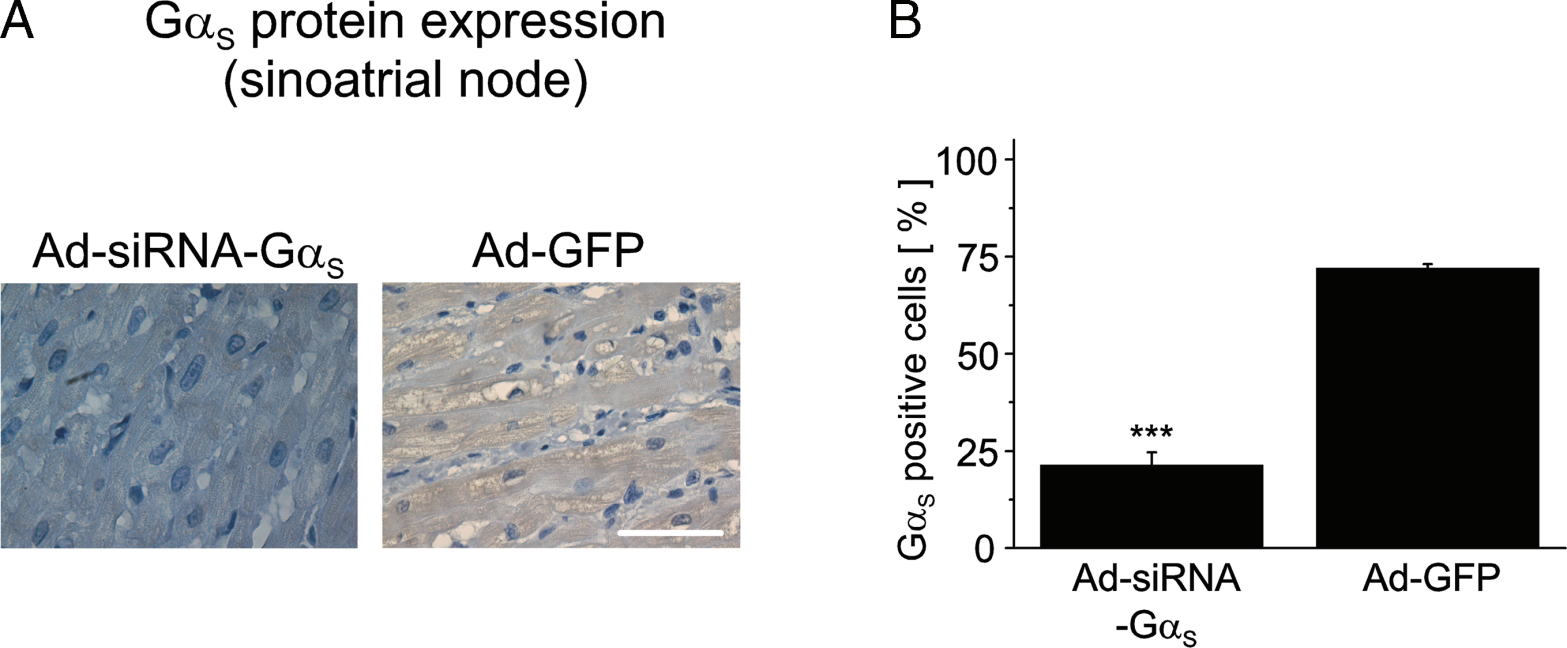
Gα_s_ protein knockdown in the sinoatrial node. Expression of Gα_s_ protein was assessed by immunohistochemistry. (A) Representative microscopic findings after treatment with Ad-siRNA-Gα_s_ and Ad-GFP (scale bar, 50 μm). (B) Quantification of Gα_s_ protein levels in *n*=5 animals per group. Data are expressed as mean±SEM (***P<0.001 vs Ad-GFP). GFP indicates green fluorescent protein.

Cardiac tissue samples were analyzed to evaluate the extent and distribution of electroporation-enhanced gene transfer (*n*=5).^10,12,13^ Quantification of GFP reporter gene expression on day 7 following Ad-GFP treatment revealed a mean expression rate of 48.1±2.4% in the targeted SAN area (Figure 3A, B). Green fluorescence signal was detected in right atria (20.1±2.6%; *P*=0.004) and left ventricles (5.4±3.4%; *P*=0.001) as well, albeit with significantly reduced efficacy compared with sinoatrial node (Figure 3A, B). Effective suppression of Gα_s_ protein in the sinoatrial node after Ad-siRNA-Gα_s_ application was demonstrated by immunohistochemistry on day 7 (Figure 4A, B). We observed a 70.5% (*P*<0.0001) decrease of Gα_s_ protein levels in the Ad-siRNA-Gα_s_ group (*n*=5) compared with control pigs receiving Ad-GFP (*n*=5), indicating successful target gene knockdown.

### Biochemical Remodeling of β-Adrenergic Signal Transduction Proteins

**Figure 5.**
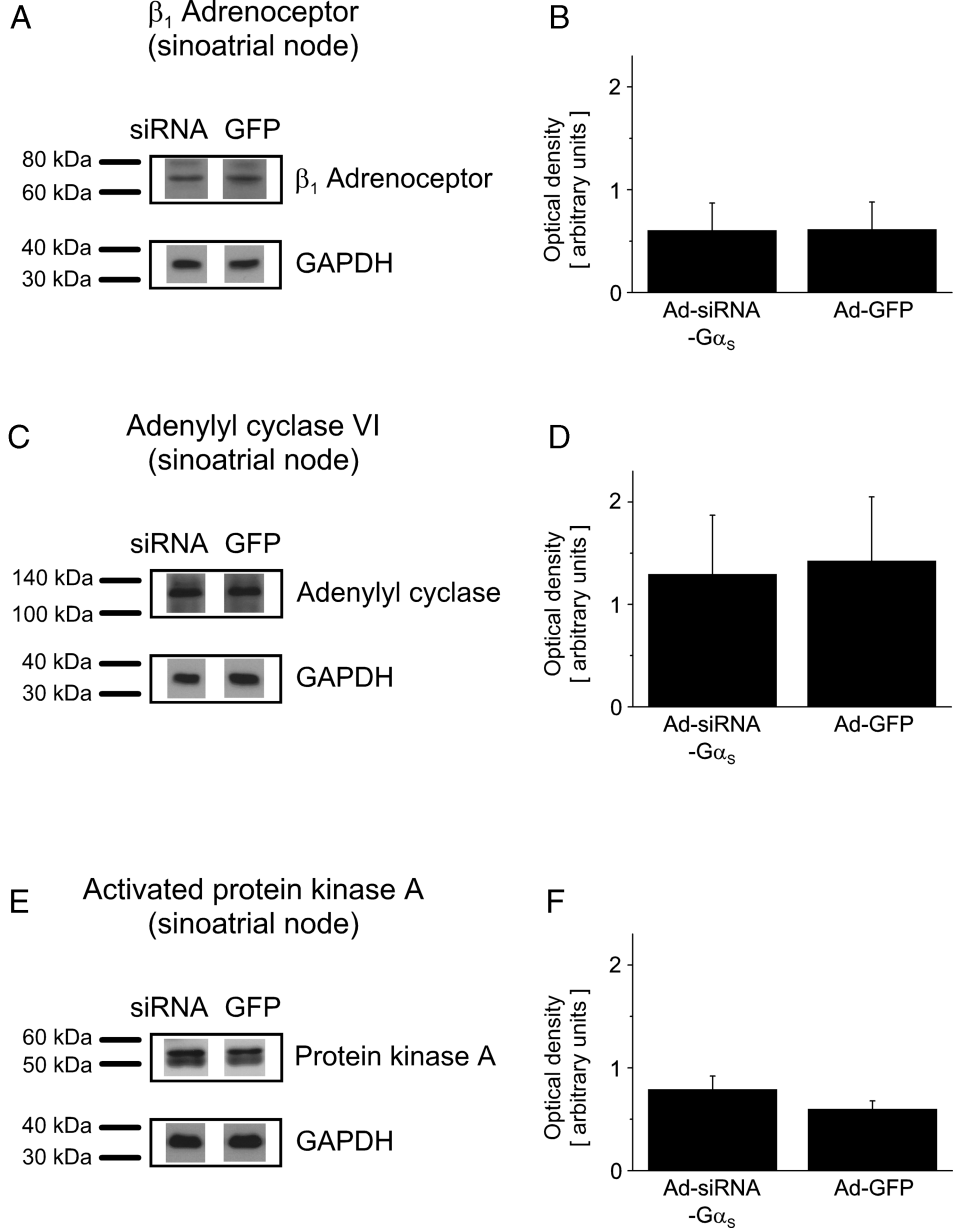
Expression of proteins involved in β-adrenergic signaling after gene therapy. Representative Western blots (A, C, E) and mean optical density (OD) values (B, D, F) are presented for study animals treated with Ad-siRNA-Gα_s_ (siRNA) and Ad-GFP (GFP), respectively (*n*=5 animals per group). Ad-siRNA-Gα_s_ treatment did not significantly affect expression of β_1_ adrenoceptors (A, B), adenylyl cyclase VI (C, D), and activated protein kinase A (E, F) in the sinoatrial node. GFP indicates green fluorescent protein.

To address the question whether reduced heart rates and Gα_s_ protein suppression were accompanied by secondary expression changes of β-adrenergic signal transduction proteins, Western blot analyses were performed on sinoatrial node tissue obtained from all study animals. We found that protein expression of β_1_ adrenoceptors (Figure 5A, B), adenylyl cyclase VI (Figure 5C, D), and phosphorylated (activated) protein kinase A (Figure 5E, F) was not affected by gene transfer.

## Discussion

### Genetic Heart Rate Control by siRNA-Mediated Gα_s_ Protein Inactivation

Increased resting heart rate has been identified as independent risk factor in cardiac disease, and heart rate-lowering treatment improved cardiovascular outcome in recent trials (BEAUTIFUL, SHIFT).^1–5^ In a subset of patients small molecule approaches are limited by reduced efficacy and by adverse effects on electrical conduction or cardiac contractility. In search for novel treatment modalities gene therapy may offer increased selectivity compared with current pharmacological therapy. Specifically, genetic modulation of β-adrenergic signal transduction through modification of G protein function in the atrioventricular node or in atrial tissue has proven effective for rate or rhythm control in atrial fibrillation animal models.^8,9,18^

In the present proof-of-concept large animal pilot study, targeted suppression of the stimulatory Gα_s_ protein in the sinoatrial node prevented heart rate increase observed in Ad-GFP animals during follow-up. Heart rates were lowered by 7.9% to 16.6% in Ad-siRNA-Gα_s_ pigs during normal sinus rhythm compared with control animals (Figure 2). Furthermore, animals receiving Ad-siRNA-Gα_s_ gene therapy exhibited attenuated heart rate increase on β-adrenergic stimulation compared with Ad-GFP controls.

### Molecular Mechanisms

Cardiac pacemaker activity is determined by activation of the *I*_f_ current and underlying hyperpolarization-activated channels (HCN) and by intracellular calcium cycling.^6,7,19–21^ HCN channel opening on membrane hyperpolarization and rhythmic Ca^2+^ release from ryanodine receptors promote membrane depolarization and initiate the cardiac action potential. Local Ca^2+^ releases stimulate Na^+^–Ca^2+^ exchange currents that accelerate diastolic depolarization in sinoatrial node cells. Triggering of action potentials is controlled by intracellular cAMP levels and protein kinase A activity. These factors increase in response to β-adrenergic stimulation and subsequent activation of stimulatory G protein α subunits, representing a basic physiological mechanism for autonomic heart rate regulation.^6,20,21^ At the molecular level the chronotropic response to β-adrenergic activation appears to be primarily mediated by modulation of Ca^2+^ cycling, whereas the basal heart rate depends on Ca^2+^- and *I*_f_-dependent mechanisms.^20,21^ The present study was based on the hypothesis that genetic inactivation of the stimulatory Gα_s_ protein and suppression of β-adrenergic activation in the SAN would provide rate control at baseline and during isoproterenol challenge.

We used an established in vivo gene transfer technique, employing local adenovirus injections in combination with electroporation to improve virus uptake into the cells.^10,12,13^ This approach resulted in 48.1% GFP reporter gene expression on day 7 in the target region (Figure 3). Furthermore, the targeted Gα_s_ protein was suppressed by 70.5% in the sinoatrial node after Ad-siRNA-Gα_s_ treatment compared with Ad-GFP controls (Figure 4), confirming gene transfer efficacy. The observation corresponds to 51.3% suppression of Gα_s_ protein expression assessed in vitro following Ad-siRNA-Gα_s_ application in HL-1 mouse atrial myocytes (Figure 1). Expression of nontargeted β-adrenergic signal transduction proteins (ie, β_1_ adrenoceptors, adenylyl cyclase VI, or phosphorylated protein kinase A) was not affected by Ad-siRNA-Gα_s_ gene therapy, ruling out any relevant compensatory remodeling within the targeted pathway (Figure 5).

In summary, we conclude that genetic suppression of Gα_s_ protein activation in the sinoatrial node decreased cardiac pacemaker activity, resulting in lowered sinus rates compared with Ad-GFP controls during follow-up and after β-adrenergic stimulation. Secondary effects of Ad-siRNA-Gα_s_ gene therapy on SAN electrophysiology by biochemical remodeling were not observed. The relative contribution of calcium cycling and of the *I*_f_ current to cardiac pacemaker function is still a matter of ongoing debate. Here, adrenergic modulation of Gα_s_ protein-associated calcium signaling is suggested as predominant target mechanism of the therapeutic approach, because heart rate reduction was observed during follow-up associated with postoperative stress and after isoproterenol application, respectively. This is consistent with recent data obtained from patients with hereditary sinus node dysfunction carrying mutated HCN4 pacemaker channels that are insensitive to the second messenger cAMP.^19^ These patients exhibited normal rate acceleration during exercise, indicating that Ca^2+^ cycling rather than HCN4 channels and *I*_f_ current determine heart rate increase during adrenergic activation.

### Clinical Implications

The present preclinical study confirms the role of Gα_s_ protein signaling in rate control during normal sinus rhythm. We further demonstrate the efficacy of gene therapy targeting Gα_s_ subunits in the sinoatrial node for rate control in a large animal model. The effect observed with Ad-siRNA-Gα_s_ therapy in pigs (7.9% to 16.6% rate reduction) is similar to pharmacological sinus rate control in humans during treatment with β blockers (13.5% to 16.4% reduction) or ivabradine (7.6% to 13.7% reduction), respectively.^1,4,22–24^ Note that values for rate reduction are provided percent to allow for ready comparison among species with different basal heart rates. Negative inotropic effects on systolic left ventricular function, a potential limitation of rate-lowering agents such as β blockers, were not observed with localized Ad-siRNA-Gα_s_ treatment. Thus, siRNA-Gα_s_ transfer may provide “exclusive” heart rate reduction similar to ivabradine.^25^ In contrast to ivabradine, however, Ad-siRNA-Gα_s_ therapy reduced the heart rate primarily during increased adrenergic activation. This mode of action is expected to be particularly beneficial in heart failure that is associated with constant and inappropriate activation of the adrenergic system. Of note, there was no case of sinus arrest, supporting the hypothesis that basal pacemaker activity was not markedly affected by Gα_s_ inactivation.

Heart rate reduction has been shown to improve clinical outcome in patients with coronary artery disease and congestive heart failure by improving coronary perfusion and through reduction of myocardial oxygen demand. Furthermore, beneficial effects of lowered heart rates on atherosclerosis have been reported.^26^ Recognizing the invasive nature of our gene delivery method, the hybrid gene application technique could currently be performed on heart failure patients during open-chest cardiac surgery required for cardiac revascularization or valve replacement. To further refine gene transfer technology, thoracotomy may be replaced in future studies by interventional, transvenous virus application.

### Limitations and Future Directions

This preclinical proof-of-concept study was designed to evaluate feasibility and short-term efficacy of biological sinus rate control using Ad-siRNA-Gα_s_ transfection in pigs. The work shares common limitations of pilot studies in large animal models including small sample size and short follow-up period. The follow-up was limited to 7 days to avoid confounding the results by loss of gene expression that occurs with first-generation adenoviral vectors. Remaining challenges of Ad-siRNA-Gα_s_ gene therapy that need to be overcome include optimized control over spacious gene distribution, proarrhythmic effects, potential tumorigenicity of vehicles and siRNA application, and prevention of local and systemic inflammatory responses. These safety issues need to be carefully addressed in larger groups of animals with extended observation periods before evaluation of antiarrhythmic gene therapy in humans. Although adenoviral vectors were used in this work owing to their ability to induce peak expression within a short time and to their high efficacy in infecting cardiac myocytes, the use of adeno-associated virus or lentivirus as vector would be more appropriate for long-term applications and to study long-term stability, efficacy, and safety of gene therapy.

## Conclusion

We demonstrate for the first time effective heart rate reduction by targeted biological modification of Gα_s_ protein signaling in the SAN. In addition, knockdown of the activating component of the β-adrenergic signaling pathway suppressed inadequate catecholaminergic heart rate increase in a large animal model. We suggest that this approach could be used as primary or supplementary treatment option in patients with cardiovascular disease after gene delivery optimization and following evaluation of long-term efficacy, safety, and toxicology.
